# Asymmetrical response of California electricity demand to summer-time temperature variation

**DOI:** 10.1038/s41598-020-67695-y

**Published:** 2020-07-02

**Authors:** Rohini Kumar, Benjamin Rachunok, Debora Maia-Silva, Roshanak Nateghi

**Affiliations:** 1 UFZ-Helmholtz Centre for Environmental Research, Leipzig, Germany; 2 School of Industrial Engineering, Purdue University, West Lafayette, USA; 3 Environmental and Ecological Engineering, Purdue University, West Lafayette, USA

**Keywords:** Climate-change impacts, Natural hazards, Energy infrastructure

## Abstract

Current projections of the climate-sensitive portion of residential electricity demand are based on estimating the temperature response of the mean of the demand distribution. In this work, we show that there is significant asymmetry in the summer-time temperature response of electricity demand in the state of California, with high-intensity demand demonstrating a greater sensitivity to temperature increases. The greater climate sensitivity of high-intensity demand is found not only in the observed data, but also in the projections in the near future (2021–2040) and far future periods (2081–2099), and across all (three) utility service regions in California. We illustrate that disregarding the asymmetrical climate sensitivity of demand can lead to underestimating high-intensity demand in a given period by 37–43%. Moreover, the discrepancy in the projected increase in the climate-sensitive portion of demand based on the 50*th* versus 90${th}$ quantile estimates could range from 18 to 40% over the next 20 years.

## Introduction

Electricity demand is influenced by many factors, including socio-demographic characteristics^4^, technology^5^, markets^6^, and climate^7–9^. Here, we focus on understanding the climate sensitivity of residential electricity demand, which is a critical factor in ensuring the resilient operation of the grid under climate change^1–3^. Recent work has isolated the effect of climate variability and change on both peak load (i.e., the highest load in a given time period) and total electricity consumption, indicating climate change will lead to greater electricity use, particularly in the residential sector^2,10–16^. This has significant implications as unanticipated increases in cooling demand in the residential sector during heat waves (i.e., periods with sustained positive temperature anomalies) can lead to unexpected supply shortages^17^, distorted electricity market prices^18,19^, as well as increased morbidity and mortality^20^, particularly in vulnerable populations and disadvantaged communities^21^.

To minimize the economic and social costs of interrupted electricity service, researchers forecast the climate-sensitive portion of residential electricity demand during extreme temperatures by harnessing methodologies from various fields, including econometrics^4,22,23^, engineering^23,24^, statistics and machine learning^13,15^. However, the existing body of literature has primarily focused on modeling the temperature response of the *central tendency* (i.e., mean/median) of the demand distribution, as opposed to considering its entire distribution^2,25–27^. We hypothesize that projections solely based on the mean/median values of the load distribution *underestimate* the climate sensitivity of high-intensity demand. Our central hypothesis is that while projections of the climate—demand nexus based on the mean/median values of demand distributions help to characterize the general trends in electricity use over time, they are likely inadequate in characterizing the climate sensitivity of the upper extremes of demand which are critical for ensuring adequate generation capacity, particularly during unusually high temperatures.

In this paper, we leverage observational data-sets to investigate the possible asymmetries in the response of electricity load to temperature anomalies. We test our hypothesis by assessing the changes in the entire distribution of daily peak electricity demand as a function of summer-time daily temperature anomalies, as observed in the three main electric utilities in California (“Methods”).

We select the state of California as a case study, as it has the largest population and economy, and ranks only second in-terms of its energy consumption in the US; rendering it a unique study region for understanding the diversity of electricity demand use patterns^28^. It is important to note that our objective here is not to develop a predictive model of electricity demand, but to investigate the potential asymmetries in the temperature response of demand and their role in future projections under climate change (“Methods”).

### Observational analysis: temperature response of electricity demand

**Figure 1:**
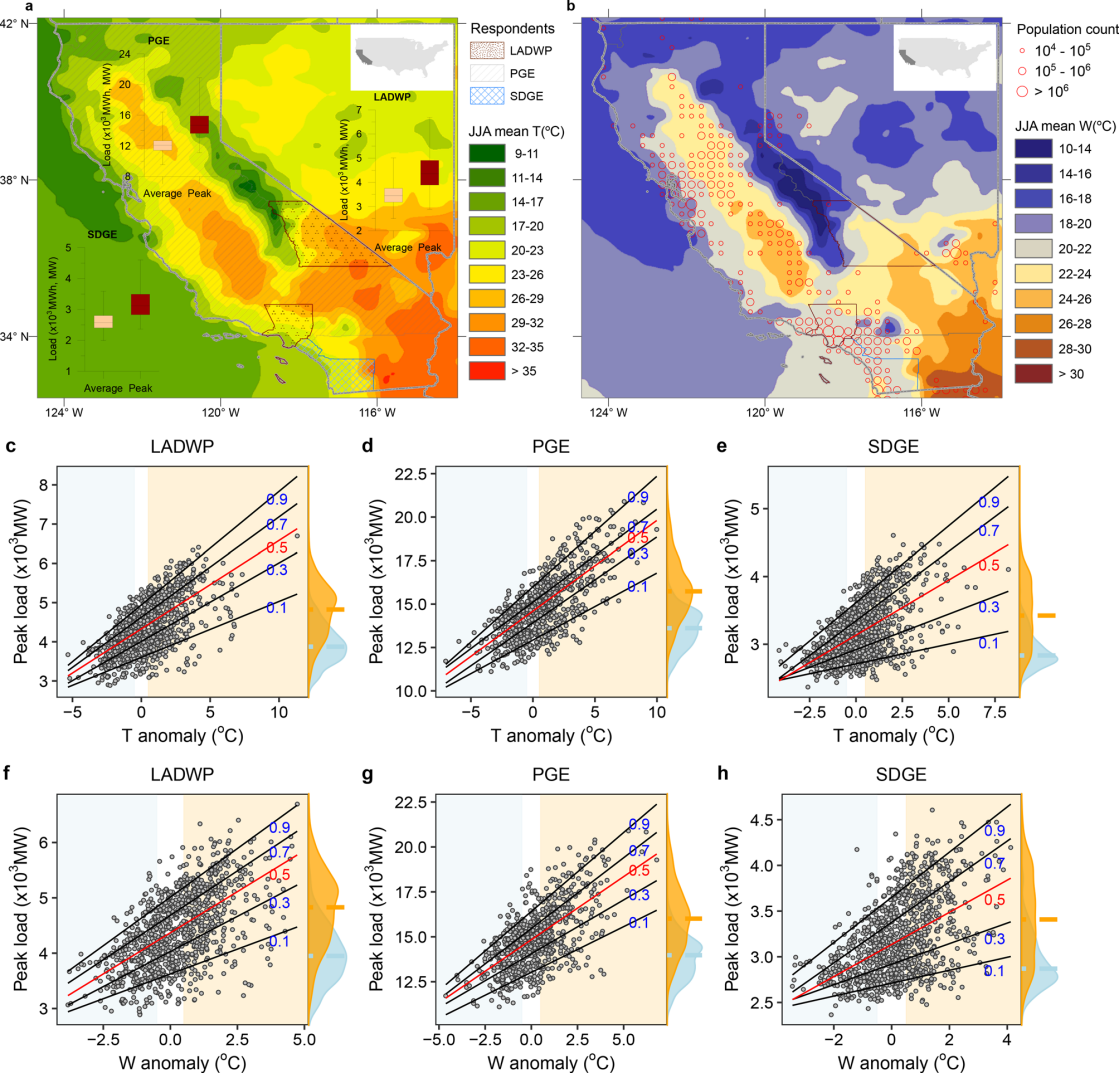
Spatial distribution of summer-time mean dry-bulb (*T*) temperature (**a**), and wet-bulb (*W*) temperature (**b**) over the study area as well as the response of peak load (**c**–**h**) to dry-bulb (*T*) anomaly (**c**–**e**) and wet-bulb (*W*) temperature anomaly (**f-h**), with quantiles ranging from 0.1 to 0.9 in increments of 0.2—the mean response is colored red. The probability density functions of temperatures for cold-anomalous (shaded blue) and warm-anomalous (shaded orange) days are shown on the right side of the graphs in **c**–**h**. The maps (**a**, **b**) were created in ArcGIS (v.10.4; https://www.esri.com/) and the line plots (**c**–**h**) were created in R (v.3.2.1; https://www.r-project.org/).

To capture how temperature anomalies influence the climate sensitive portion of electricity demand, we use daily peak load and daily average electricity consumption during the months June, July, and August (JJA) in the years 2006–2016 from the three main Californian utilities: Los Angeles Department of Water and Power (LADWP) , Pacific Gas and Electric Company (PGE), and San Diego Gas and Electric Company (SDGE) (Fig. 1a,b). These three utilities combine to provide electricity to over 95% of Californians^30^. We consider the daily variation of both dry-bulb (*T*) and wet-bulb (*W*) temperatures in our analysis, following previous studies showing the importance of *T* and humidity (embedded in *W*), affecting electricity consumption patterns across US^3^. Both climatic variables are aggregated to an equivalent geographic scale of the electricity data using population as a spatial weighting factor (“Methods”). Daily temperature anomalies are calculated as the difference between a daily temperature value and the respective long-term mean over the same time period (see “Methods” for more details). For example, a temperature anomaly of 1 $^\circ$C over a utility region for a given day indicates a deviation of 1 $^\circ$C above the respective daily long-term mean, estimated for that specific region. Figure 1a,b depicts the spatial distribution of population (2010), the mean JJA daily dry-bulb (*T*) and wet-bulb (*W*) temperatures ($^{\circ }$C), and the distribution of the daily peak load (MW) and daily average consumption (MWh) over the three utility regions for the observational period 2006–2016.

To analyze the varying response of the daily peak load and daily average electricity consumption as a function of temperature anomaly, we leverage quantile regression (“Methods”). Quantile regression relates the conditional percentiles of the load to the temperature anomalies, allowing for the comparison of, for example, the 90th percentile versus the 50th percentile of load as a function of varying temperature anomalies^31^. Figure 1c–h shows the trends—as fitted regression lines—for various percentiles (i.e., 0.1–0.9) of the daily peak load as a function of dry-bulb (*T*; c–e) and wet-bulb (*W*; f–h) temperature anomalies for all three utilities analyzed in this study, representing the temperature response of residential electricity demand. In line with previous findings^2^, there is a positive correlation between temperature and summer electricity demand as well as significant variability in the residential electricity demand associated with warm-anomalous days. A similar trend of energy consumption to varying temperature anomaly is also observed for the daily average electricity consumption, consistently across all three utility regions (see Supplementary Figure S1)—indicative of a rather general response behavior of increasing electricity demand in warm-anomalous days and higher climate sensitivity of upper extremes.

**Figure 2:**
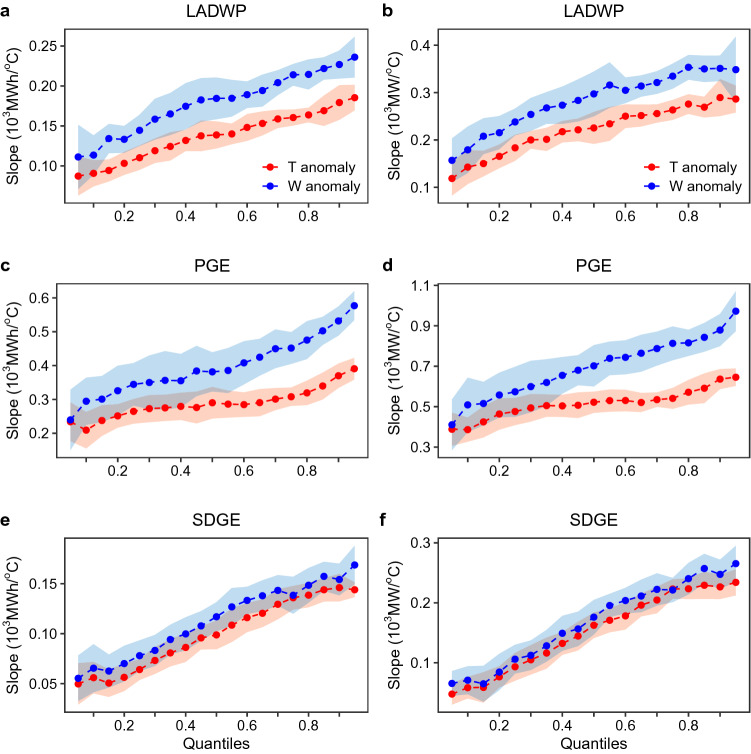
Quantile regression model outputs indicating asymmetry in temperature response of average daily load (**a**, **c**, **d**) and daily peak load (**b**, **d**, **f**) to dry-bulb temperature anomaly (*T*; red) and wet-bulb temperature anomaly (*W*; blue) by quantile (ranging from 0.1 to 0.9 in increments of 0.1). Values on the Y-axes represent the slope estimates of the quantile regression models in units of $10^3$ Mega-Watts (MW) or Mega-Watts hours (MWh) per degrees Celsius ($^\circ$C). The shaded area represents 95% confidence interval. Figure was created in R (v.3.2.1; https://www.r-project.org/).

The response of both average daily consumption (Fig. 2a,c,e) and daily peak load (Fig. 2b,d,f) to dry-bulb temperature anomaly (*T*; red lines) and wet-bulb temperature anomaly (*W*; blue lines) are shown in Fig. 2. Specifically, the temperature responses are measured as the slopes of the regression lines depicted in Fig. 1c–h. In the case of peak daily load response to dry-bulb temperature (*T*) anomaly, the slopes increase from 0.14, 0.39, 0.06 (10$^3~$MW/$^\circ$C), to 0.29, 0.64, 0.23 (10$^3~$MW/$^\circ$C) from the 10th to the 90th percentiles for LADWP, PGE, and SDGE respectively (Fig. 2b,d,f); representing a 103%, 65.1%, and 288% increase. These results illustrate the difference in the climate-sensitivity of daily peak load to temperature anomalies at the upper and lower extremes. Importantly, the upper extremes of peak load are substantially more sensitive to temperature anomalies, as evidenced from steeper slopes present at higher quantiles, indicating warm-anomalous temperatures will have a disproportionate impact on higher-intensity electricity consumption. Moreover, the asymmetrical response of load to anomalies is much more pronounced when considering daily anomalies of wet-bulb (*W*) temperature (a combined measurement of heat and humidity) than that of dry-bulb (*T*) temperature, as observed by the steeper slopes of the blue lines in Fig. 2. Analogous to the daily peak load behavior, the response (slope) of average daily load to temperature anomalies increases with higher quantiles (Fig. 1a,c,e).

As power reserve margins (i.e., the buffer capacity to supply summer-time peak load) are designed by considering the upper limits of electricity use, it is critical to note the difference between the climate sensitivity of the 90th percentile versus the mean/median values of peak load (which are commonly used for capacity margin calculations). The median peak daily load responses to temperature anomaly—as indicated by the slopes of the quantile regression at the 50th percentile (Fig. 2)—are 0.23, 0.52, 0.16 $\times$ 10$^3~$MW/$^\circ$ C as compared to 0.29, 0.64, 0.23 $\times$ 10$^3~$MW/$^\circ$C at the 90th percentile for LADWP, PGE, and SDGE respectively, representing a 28.4%, 21.9%, 39.5% increase. These substantial differences in the climate-sensitivity of the 90th percentile versus median load to temperature anomalies imply that the current practice does not adequately capture the higher temperature sensitivity of the upper tails of load corresponding to high intensity users. Therefore, planning reserve margins based on existing approaches will likely underestimate requisite levels of excess capacity to minimize the risk of rolling outages during temperature extremes^32^.

### Future temperature response of electricity demand

**Figure 3:**
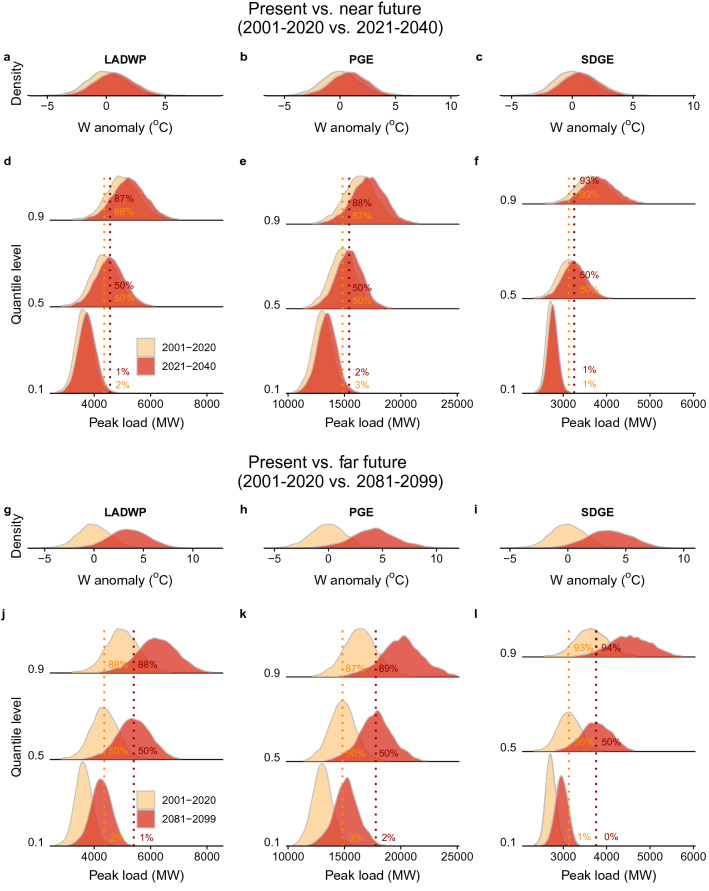
Illustrating the asymmetrical temperature response of electricity demand under future climate change scenarios. The distributions of wet-bulb (*W*) temperature anomalies are shown for the three utility service areas in **a**–**c**. The distribution of base period (2001–2020) peak loads (shaded yellow) and projections of near future period (2021–2040) (**a**–**f**) and far future period (2081–2099) (**g**–**l**) peak loads (shaded red) under RCP8.5 for the three utilities of LADWP, PGE, and SDGE are shown. Vertical dotted lines in **d**–**f**, **j**–**l** show how the median value of the 50th percentile model would fall relative to the median values of the 10th percentile and 90th percentile models. For example, in **d**, 88% of the daily peak load predicted by the 90th percentile model exceeds the median value predicted by the 50th percentile model during the base period, representing a 35% increase. Figure was created in in R (v.3.2.1; https://www.r-project.org/).

To understand the implications of the asymmetrical temperature response of energy demand under future climate conditions, we use climate projection data extracted from five Global Circulation Models over a base (or reference) period (2001–2020), near future period (2021–2040), and far future period (2081–2099) (“Methods”). Specifically, we leverage the daily June, July, August temperatures over the two time periods, using the RCP8.5 (Representative Concentration Pathway) climate scenario^33^, representing global temperature changes corresponding with high future greenhouse gas emissions. Similar to the observational analysis, we calculate the daily temperature anomalies in each climate model space separately that represent the daily deviation of (dry and wet-bulb) temperature in future periods with respect to corresponding daily mean values estimated over the reference period (see “Methods” for more details). For this illustration propose, we then formulate three models of future electricity use for each utility based on the corresponding quantile regression results of the observed temperature response of daily peak load at the 10th , 50th, and 90th percentiles. We refer to these as the 10th , 50th, and 90th percentile models, used to project the respective peak daily electricity load in response to future temperature anomaly. The corresponding distributions of the daily peak loads along with the temperature anomalies for the considered two future periods are shown in Fig. 3. We reiterate that our aim here is to demonstrate the implications of the asymmetrical temperature response of the climate sensitive portion of demand and not to build a high-fidelity demand prediction model which would require information about technological and demographic changes. Our analysis results underscores the importance of considering this asymmetrical response behavior that will likely improve the accuracy of electricity demand forecasts.

**Table 1:** Mean and standard deviation of projected daily peak load values (MW) estimated for near and far futures for each region of study and at 0.1, 0.5, and 0.9 quantiles.

Quantile level	Period		Region-wise Peak load [MW]		
			LADWP	PGE	SDGE
0.1	Present	2001–2020	3623.84 (305.32)	13026.81 (878.63)	2710.98 (111.64)
	Near future	2021–2040	3745.03 (322.01)	13436.25 (896.53)	2761.72 (119.17)
	Far future	2081–2099	4246.66 (372.04)	15137.31 (1125.64)	2961.67 (142.73)
0.5	Present	2001–2020	4366.52 (506.39)	14846.20 (1212.47)	3134.37 (277.50)
	Near future	2021–2040	4567.52 (534.07)	15411.21 (1237.17)	3260.50 (296.23)
	Far future	2081–2099	5399.48 (617.04)	17758.59 (1553.32)	3757.50 (354.78)
0.9	Present	2001–2020	5010.62 (598.47)	16419.02 (1517.96)	3649.74 (390.21)
	Near future	2021–2040	5248.16 (631.18)	17126.40 (1548.88)	3827.09 (416.56)
	Far future	2081–2099	6231.38 (729.23)	20065.21 (1944.69)	4525.96 (498.88)

Values are calculated using simplified wet bulb temperatures, means are listed with standard deviations in parentheses. Note, these values correspond to daily estimates of the distributions shown in Fig. 3.

Comparing the distributions of the daily peak loads for the 50th and 90th percentile models in each period helps determine how frequently the 50th percentile model will underestimate the 90th percentile of the *climate-sensitive* portion of the load. Results indicate a significant gap between the 50th and 90th percentile daily peak load in all periods under study. Specifically, high-intensity daily peak load is *underestimated* across all periods (i.e., baseline, near future and far future) by 37–38%, 37–39%, and 43–44% in the respective utilities when using a median-based rather than 90th percentile model (Fig. 3). The summary statistics of the daily peak load (in MW)—corresponding to the 0.1, 0.5, and 0.9 quantile models—in response to wet-bulb temperature (*W*) anomalies for each time period and in each utility region are shown in Table 1.

The difference between the estimated daily peak load during present and near future periods, as well as the difference between present and far future periods increases as higher quantilies are considered (see Table 1). For example, in the LADWP region, the difference between the present and near future daily peak loads increases from 201 MW in the 0.5 quantile to 238 MW in the 0.9 quantile, an increase of 18%. In other words, the discrepancy in projected increase between the 0.5 and 0.9 quantile model could range from 18% (LADWP) to 40% (SDGE). This pattern appears across both (near- and far-future) time periods, demonstrating that disregarding the asymmetry in temperature response of demand will lead to underestimating the climate-sensitive portion of the upper extremes of demand, regardless of the future planning horizon. This also holds for the results associated with dry-bulb (*T*) temperature anomalies as shown in Supplementary Table 1.

Figure 3d also indicates that approximately 88% (87%) of daily peak load values projected by the 90th percentile model in LADWP are greater than the median of the 50th percentile model over the base (near future) periods. These underestimation values for the PGE and SDGE are in the order of 87% (88%) and 93% (93%) respectively (Fig. 3e, f). These results remain remarkably consistent if evaluating temperature anomalies using alternative formulations i.e., based on the dry-bulb (*T*) temperature anomalies (see Supplementary Figure S4).

## Discussion and concluding remarks

In this exploratory work, we provide observational evidence of an asymmetric (summer-time) temperature response of electricity demand and consumption. By isolating the impact of climate variability and change on both daily peak load and average daily consumption, we quantify the temperature responses of electricity demand across three utilities in the state of California. We find significantly higher levels of climate sensitivity in high intensity demand (both daily peak load and average daily consumption) under both current and future climate scenarios when evaluating both wet-bulb and dry-bulb temperatures despite California’s unique load structure known colloquially as the duck curve. We emphasize that this asymmetry is present empirically in the demand profile of California, but further studies are needed to investigate this trend in other regions of the US or the world. Furthermore, this asymmetry has significant implications for designing adequate power reserve margins to ensure the resilient operation of the grid during extreme temperatures.

We emphasis that the projections provided for the period 2081–2099 should not be considered a climate change impact assessment as conducted in prior studies^14,34^. Instead, the projections are provided to demonstrate how the observational evidence of this asymmetry may bear out under future climate conditions. We therefore propose accounting for the asymmetrical temperature response of demand in addition to other key factors such as technology trends, as well as shifts in economic and demographic characteristics which affect projected electricity use.

Record summer temperatures regularly lead to unanticipated electricity demand across many parts of the country, distorting electricity market prices and inducing rolling outages. Lack of consideration of the non-symmetric temperature response of peak and average electricity load underestimates the climate sensitivity of high intensity demand and severely threaten the resilience of the grid. The impacts of service disruptions often disproportionately affect the disadvantaged communities. This is because high-intensity energy users do not necessarily belong to affluent communities for various reasons such (a) higher occupancy rates to save rental expenses^35^, (b) residence in crime-areas (inability to open windows for ventilation), and (c) residence in neighborhoods with higher heat-island effects^36–39^. Therefore, the fallacy of symmetric temperature response of electricity demand has significant environmental justice implications. A failure to consider the increased temperature response of high-demand users will drive future inequity in access to the reliable provision of electricity.

## Methods

### Peak and average electricity demand

To characterize the non-symmetric response of both daily peak and daily average electricity loads to temperature anomalies, we aggregate hourly summer (June, July, August) peak load and average electricity consumption for the state of California during 2006–2016. Peak and average electricity loads are taken from three California Utilities: Los Angeles department of Water and Power (LADWP), Pacific Gas and Electric (PGE), and San Diego Gas and Electric (SDGE); available from the US Energy Information Administration (EIA) public reports. In the analysis results presented in the main text (Fig. 1c–h), we focus on the observed temperature response of daily peak residential electricity demand. However, a similar trend of asymmetric climate sensitivity of load is also observed when analyzing daily average electricity consumption (see Supplementary Figure S1).

### Temperature anomalies

The daily dry-bulb (*T*) and wet-bulb (*W*) air temperature anomalies are calculated for the summer months of June, July, and August in the time period corresponding with the electricity demand data. The required climatic variables (i.e., near-surface air temperatures, pressure and humidity fields) were obtained from the NCEP North American Regional Reanalysis (NARR)^40^ which is available at an approximately 32 km spatial resolution since the beginning of 1979. We aggregate the climate variables to the utility level taking the population as the spatial weighting factor, which in line with previous studies focusing on residential electricity load^2, 15^. We used the 2010 UN-adjusted Gridded Population of the World dataset (Version 4) for the study area that was obtained from Socioeconomic Data and Applications Center (SEDAC; http://sedac.ciesin.columbia.edu). This step results in an aggregated estimates of the daily climate variables for the period 2006–2016 corresponding with the resolution of our electricity production values. To test the sensitivity of our results to underlying climate database, we additionally compared the values using the gridded surface meteorological dataset (gridMET)^41^ and WATCH forcing data methodology applied to ERA-Interim data (WFDEI)^42^ and found similar results (see Supplementary Figures S2 and S3). Results reported here in the main text are based on the NARR derived climate datasets.

We calculate the daily anomalies as the deviation between a daily average temperature value from the corresponding long-term mean values estimated over the 30-years time-period (1981–2010). The long-term mean for each calender summer day is estimated by averaging all values that fall within a window of $\pm 7$ days centered on that calendar day; thus allowing us to account for temporal variation in climatic variables. We also performed additional analysis to check the robustness of our findings to the chosen (30-years) baseline period for estimating the daily temperature anomalies. To this end, we calculated the daily anomalies with respect to the 11-years baseline period (2006–2016), which is consistent with the availability of the electricity demand datasets; and found similar results for the asymmetrical response behavior of the daily (peak and average) energy demands to temperature anomalies across the study regions (see Supplement Figures S5 and S6). In our analysis, we focus on the sensitivity of electricity demand to both dry-bulb and wet-bulb air temperature anomaly. Here, we consider the formulation of the simplified wet-bulb globe temperature (*W*) as described in Ref.^43^. While not included in this manuscript, we also tested other formulations of climatic indicators accounting for both dry-bulb air temperature and relative humidity such as National Weather Service based heat stress or discomfort index^43^ and found similar asymmetric response of the electricity loads.

### Quantile regression

For each utility, we perform quantile regression (QR) between daily temperature anomalies and corresponding (peak and average) energy demand/consumption. Quantile regression is a form of regression modeling in which conditional *quantiles* of the response variable are estimated^31^, allowing for a richer characterization of the data by allowing for evaluation of the impact of a covariate across the entire distribution of the response variable rather than just its mean^44^. The coefficients of each quantile regression model can be interpreted similarly to the coefficients of an ordinary least squares regression. Quantile regression has previously been utilized to address asymmetries and heteroskatasticity in electricity demand data^36,45,46^.

In this work, we use quantile regression to generate estimates of the conditional quantiles of the conditional quantiles of electricity demand as a function of temperature anomalies. For the demonstration of asymmetrical behavior, here we use a linear model for the conditional quantiles—as shown in Fig. 1 by the fitted lines. Hot and cold anomalies are calculated as values which exceed 0.5 $^\circ$C above or below average respectively. Distributions for the hot and cold anomalous regions are also shown in Fig. 1. Slopes of the respective quantile regressions (representing the regression coefficients for each percentile) are shown in Fig. 2 along with a 95% confidence interval band calculated through bootstrap sampling.

### Climate projections

To investigate the impact of the asymmetric temperature response of demand under future climate scenarios, we use the developed quantile regression models to estimate future daily peak and daily average electricity values based on climate projections at the 10th , 50th, and 90th percentiles. Climate projections are taken from the archive of five CMIP5 datasets: Geophysical Fluid Dynamics Laboratory Earth System Model 2M (GFDL-ESM2M)^47^, Hadley Global Environment Model 2-Earth System (HadGEM2-ES)^48^, Institut Pierre Simon Laplace Earth System Model for the 5th IPCC report (IPSL-CM5)^49^, Japan Agency for Marine-Earth Science and Technology Earth System Model (MIROC-ESM-CHEM)^50^, and the Norwegian Earth System Model (NorESM1-M)^51^. These global climate datasets are available from the Inter-Sectoral Impact Model Intercomparison Project^52^ at a 0.5 degree spatial resolution. To illustrate the magnitude of future climate impact, we use the daily outputs of the climate simulations under the RCP8.5 (Representative Concentration Pathways) scenario and contrasted the summer months (JJA) daily peak load/average consumption for two future periods: 2021–2040 (near-future) and 2081–2099 (far-future)—with respect to a baseline (or reference) period of 2001–2020. Similar to observational analysis, we first estimated the utility-wide values of daily dry-bulb and wet-bulb temperature during the summer months (JJA), and then calculated the daily anomalies using the respective daily long-term mean calendar day estimates (accounting for the $\pm 7$ days window) of the baseline period for each climate model and utility region separately. For each utility region (LADWP, PGE, and SDGE), temperature type (*T* and *W*), and future period (2021–2040 and 2081–2099), we then pool up the daily temperature anomalies from all five GCMs together and apply the corresponding (region and temperature specific) quantile regression (QR) model to obtain the estimates of respective energy load/consumption for different quantile levels (e.g., 0.1, 0.5 and 0.9).

## Supplementary information

Supplementary file1 (PDF 724 kb)

## Data Availability

NCEP Reanalysis data provided by the NOAA/OAR/ESRL PSD, Boulder, Colorado, USA, from their Web site at https://www.esrl.noaa.gov/psd/. California electricity demand data available upon request; and can be accessed from https://www.eia.gov/electricity/data/eia861/ and http://www.caiso.com/Pages/default.aspx. Gridded Population data can be assessed from SEDAC http://sedac.ciesin.columbia.edu; and the climate model datasets from ISI-MIP https://www.isimip.org.
