# Supplementary material for: Asymmetrical response of California electricity demand to summer-time temperature variation
Source: Sci Rep. 2020 Jul 2;10:10904. doi: 10.1038/s41598-020-67695-y (PMC7331730; doi:10.1038/s41598-020-67695-y)
Supplement: Supplementary file 1 — Supplementary file1 (PDF 724 kb) [file 41598_2020_67695_MOESM1_ESM.pdf]

Supplementary Material for

Asymmetrical response of California electricity demand to summer-time temperature variation

Rohini Kumar[1,*,+], Benjamin Rachunok[2,+], Debora Maia-Silva[3], and Roshanak Nateghi[2,3,*,+]

[1]UFZ-Helmholtz Centre for Environmental Research, Leipzig, Germany

[2]School of Industrial Engineering, Purdue University, USA

[3]Environmental and Ecological Engineering, Purdue University, USA

[*]rohini.kumar@ufz.de; rnateghi@purdue.edu

[+]Authors with equal contributions

Supplemental Figure S1 shows the distribution of temperature anomalies from NARR data[1] compared to *average load* for each utility in the state of California. Results presented in the main text show *peak load*. In Figure S1 we see similar asymmetries in the temperature response of electricity demand across different percentiles. Supplemental Figures S2 and S3 shows the relationship between (peak and average) electricity load and temperature anomalies based on different climate data sources, gridMET[2] and WFDEI[3] respectively. Supplemental Figure S4 shows the sensitivity of daily peak load projections to the changes in the dry-bulb temperature anomalies following the same methodology as in Figure 3 in the main text. Additionally the Table S1 shows the corresponding mean and standard deviation of projected changes corresponding to Figure S4 (similar to information of Table 1 in the main text). Similar to Figures 1 and 2 described in the main text, supplemental Figures S5 and S6 report the results of quantile regression analysis based on the daily temperature anomalies using data of 2006-2016 period only (also for the estimation of long-term mean values) – the 11-year time-period in which both climate and energy demand data are commonly available across the study region.

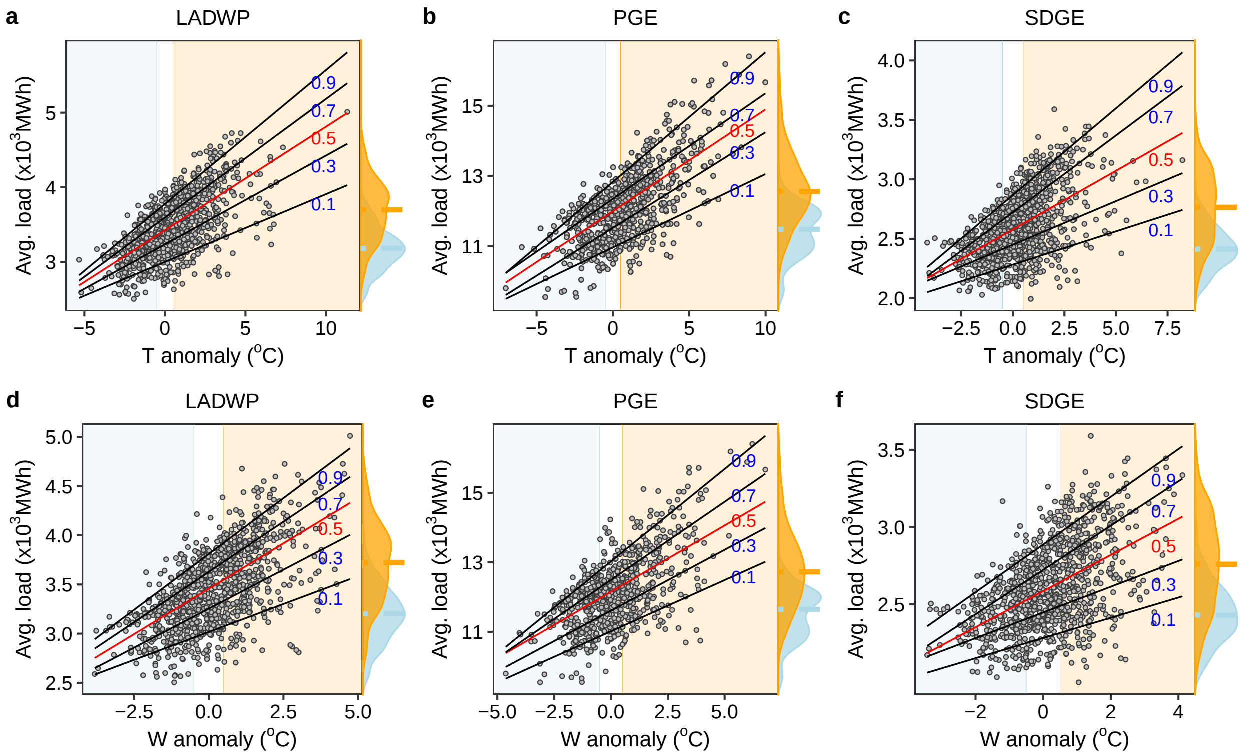

Figure S1: Daily average electricity load and temperature anomalies evaluated for quantiles across three Californian utilities. This is similar to Figure 1 in the main text, but here we compare the daily average load against both dry and wet bulb-temperature anomalies. Figure was created in in R (v.3.2.1; `www.r-project.org/`).

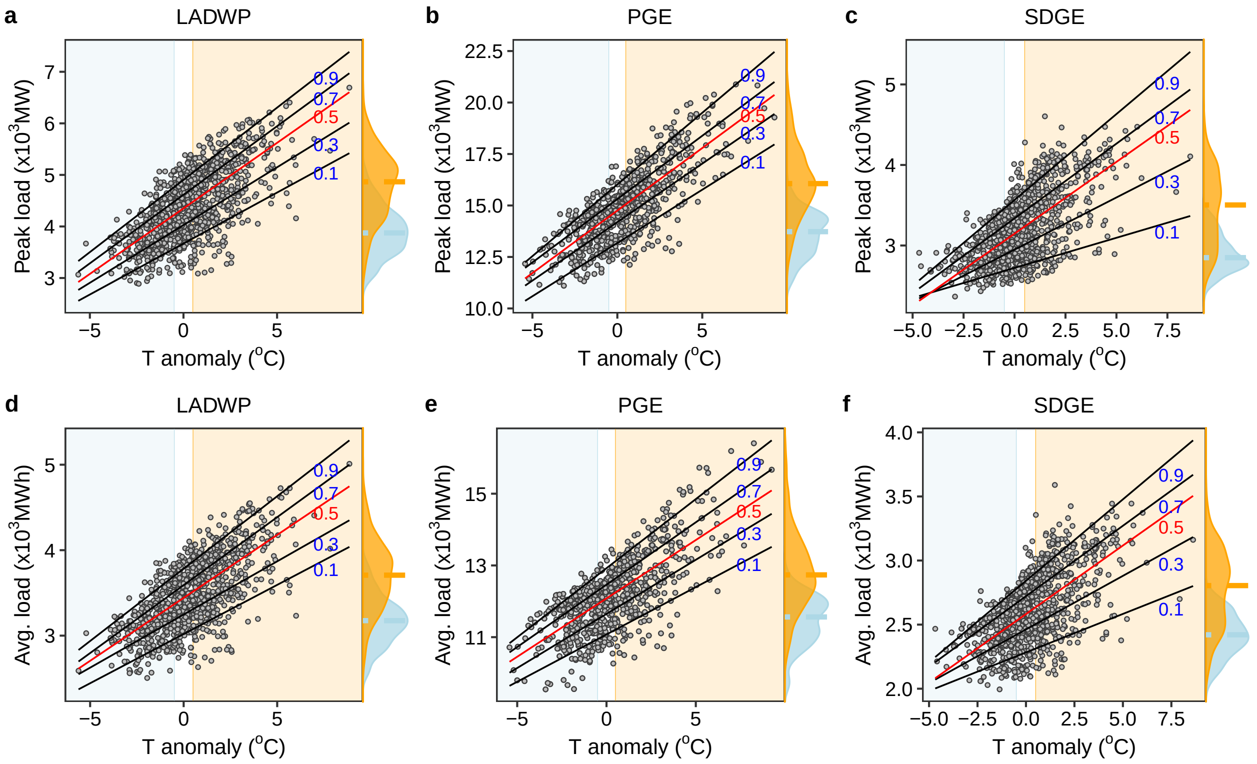

Figure S2: Daily peak and average electricity load and temperature anomalies evaluated by quantile using an alternative source of temperature data. Daily air temperature data in this figure comes from gridMET[2]. Figure was created in in R (v.3.2.1; `www.r-project.org/`).

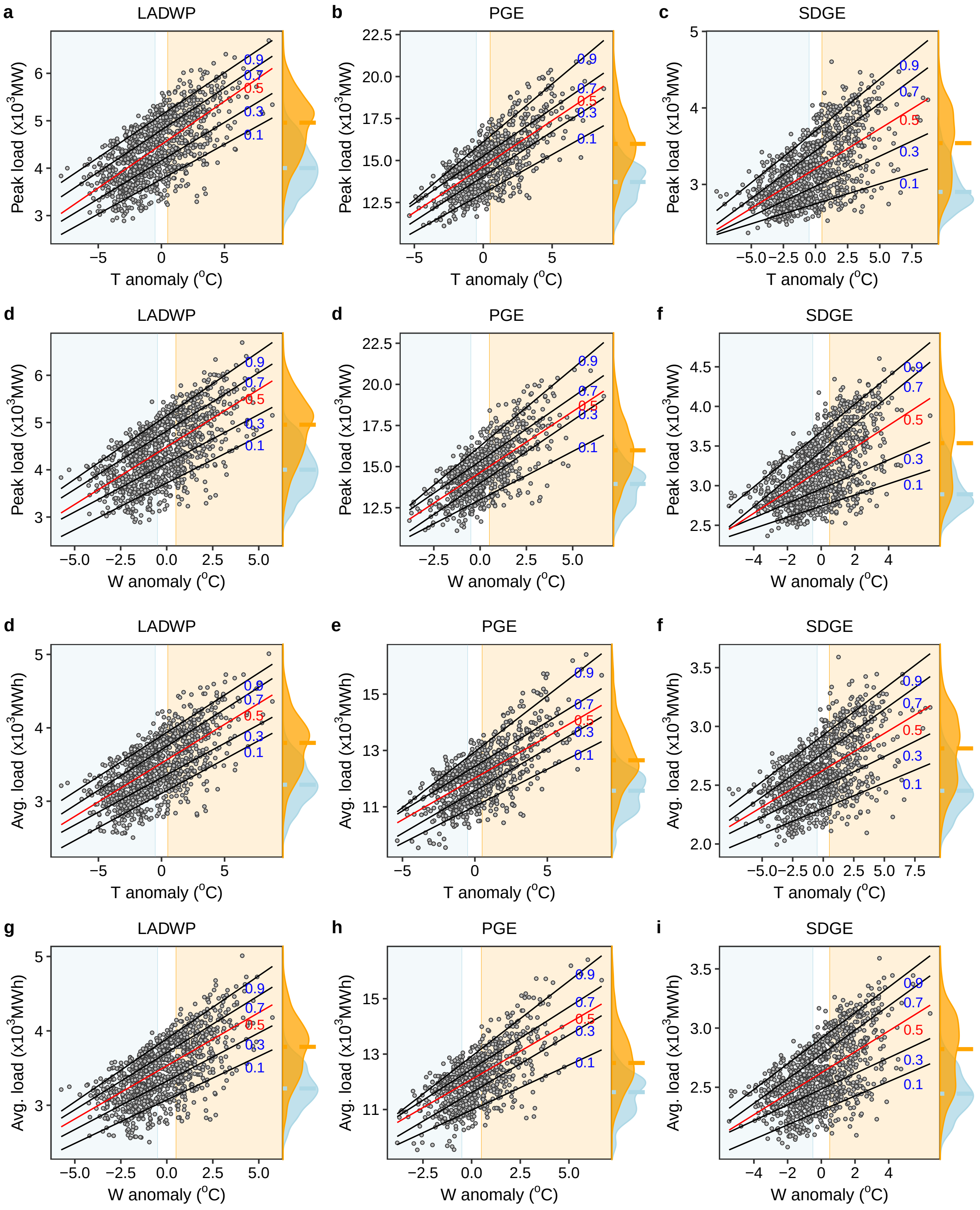

Figure S3: Similar to Figure 1 of the main text and Figure S1 (here) but using the temperature anomalies based on an alternative WFDEI[3] dataset. Figure was created in in R (v.3.2.1; `www.r-project.org/`).

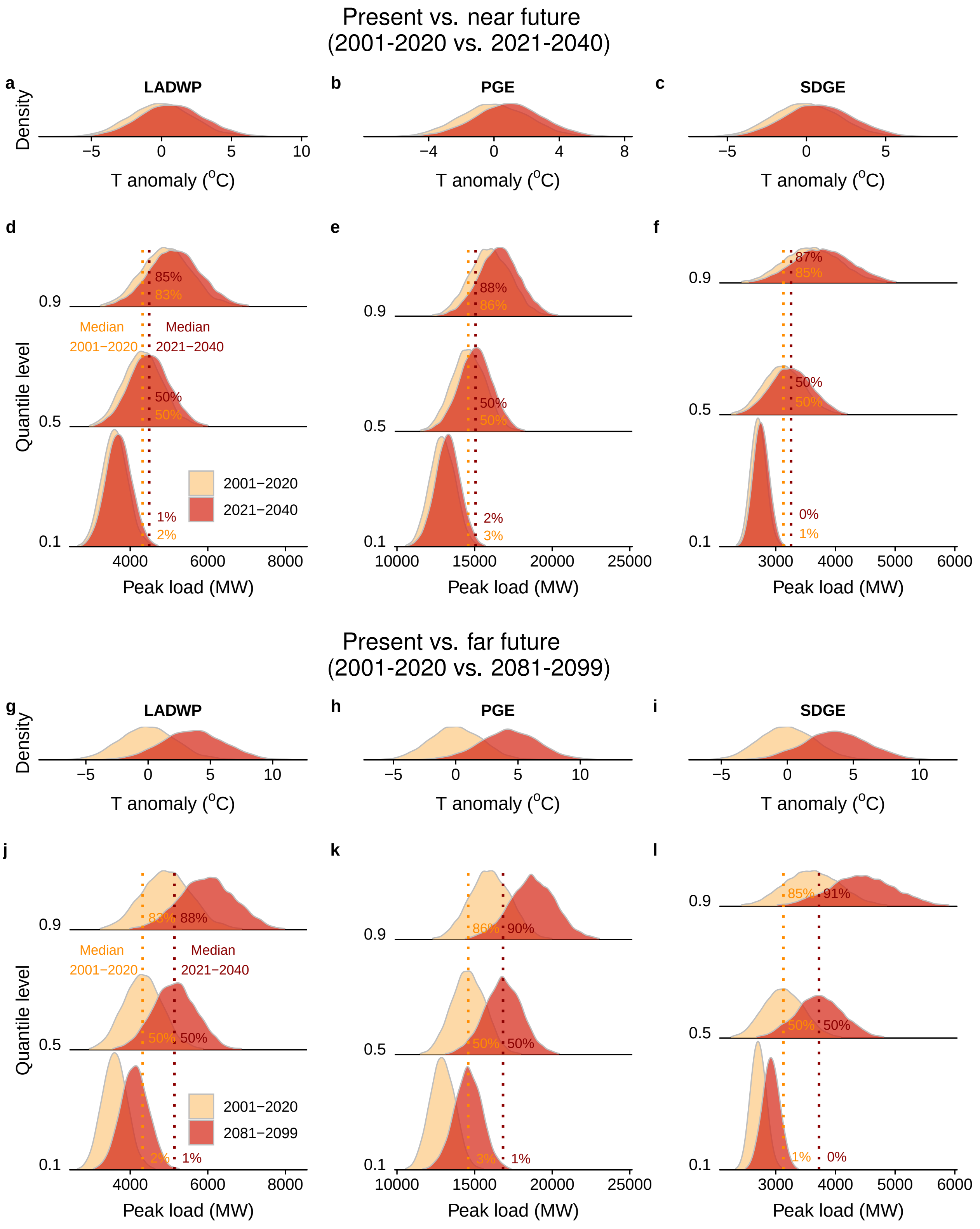

Figure S4: Similar to Figure 3 in the main text for the projections of the daily peak load, but based on anomalies using the dry-bulb temperature. Figure was created in in R (v.3.2.1; `www.r-project.org/`).

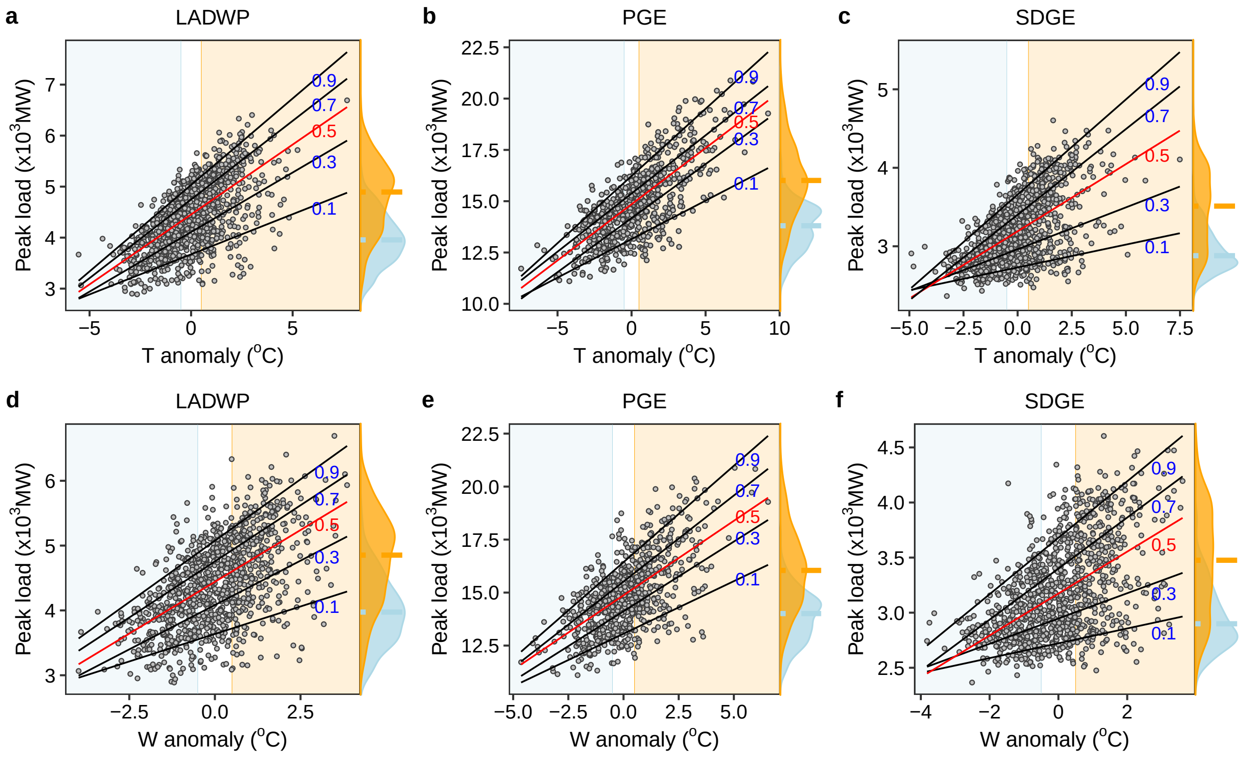

Figure S5: Similar to Figure 1 in the main text for the quantile regression analysis, but based on daily temperature anomalies estimated using data of 11-years (2006-2016). Figure was created in in R (v.3.2.1; `www.r-project.org/`).

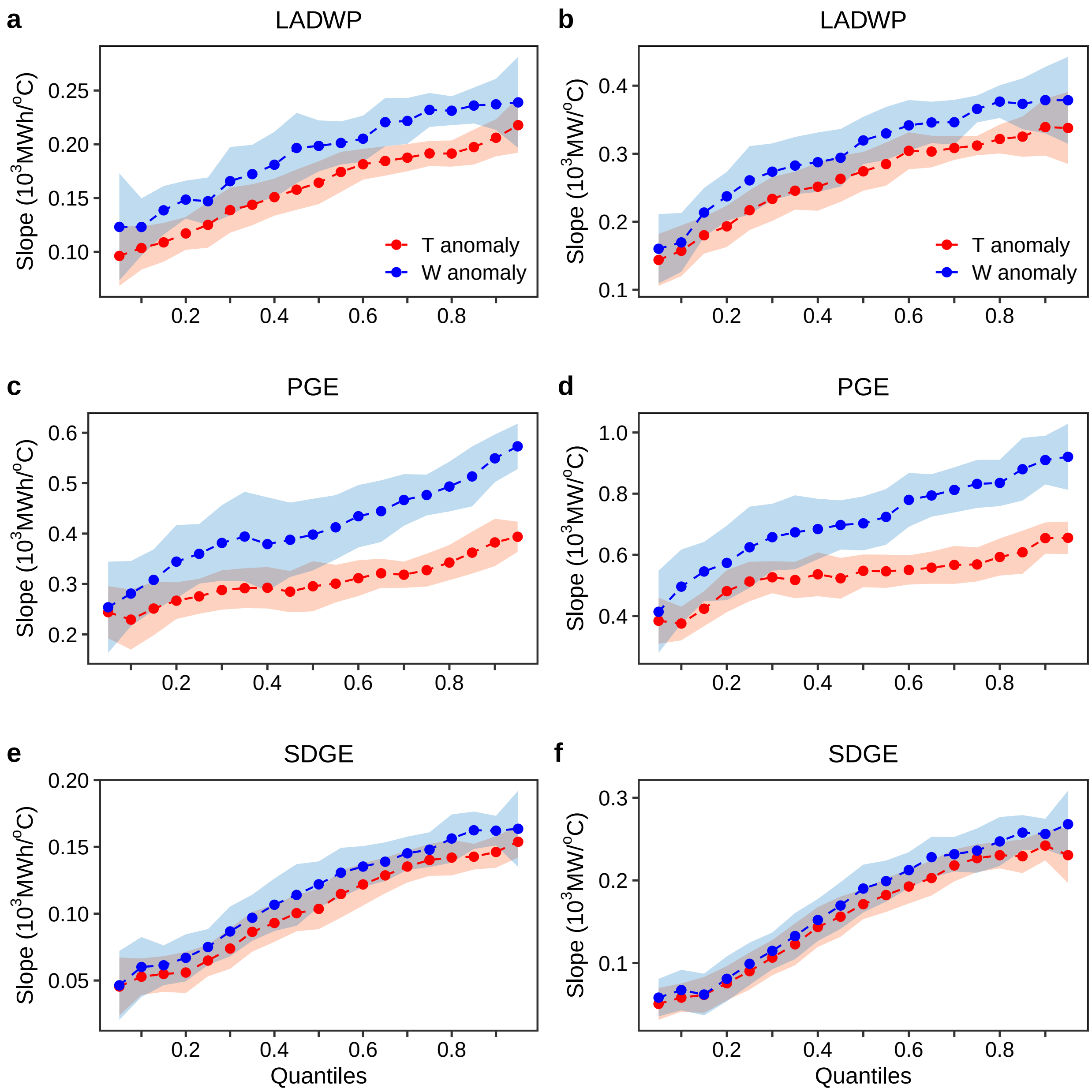

Figure S6: Similar to Figure 2 in the main text for the estimated slopes of quantile regression models, but based on daily temperature anomalies estimated using data of 11-years (2006-2016). Figure was created in in R (v.3.2.1; `www.r-project.org/`).

Table S1: Mean and standard deviation of daily peak load values (MW) estimated for three time-periods for each region of study and at 0.1, 0.5, and 0.9 quantiles. Values here correspond to the anomalies of daily average dry bulb temperatures, means are listed with standard deviations in parentheses. This is a complementary to information given in Table 1 in the main text.

Quantile level	Period		Region-wise Peak load [MW]		
			LAWP	PGE	SDGE
0.1	Present	2001-2020	3601.61 (319.20)	12931.31 (804.71)	2710.30 (121.88)
	Near future	2021-2040	3707.52 (329.56)	13267.62 (811.19)	2755.92 (126.33)
	Far future	2081-2099	4117.25 (359.36)	14581.75 (927.013)	2924.61 (143.44)
0.5	Present	2001-2020	4328.91 (504.05)	14603.53 (1086.72)	3135.02 (338.81)
	Near future	2021-2040	4496.14 (520.40)	15057.7 (1095.47)	3261.83 (351.17)
	Far future	2081-2099	5143.14 (567.45)	16832.35 (1251.88)	3730.74 (398.73)
0.9	Present	2001-2020	4938.58 (647.40)	16003.04 (1325.59)	3615.24 (472.70)
	Near future	2021-2040	5153.37 (668.40)	16557.04 (1336.27)	3792.16 (489.94)
	Far future	2081-2099	5984.38 (728.84)	18721.78 (1527.05)	4446.37 (556.31)

1 References

1. Mesinger, F. *et al.* North american regional reanalysis. *Bulletin of the American Meteorological Society* **87**, 343–360 (2006).

2. Abatzoglou, J. T. Development of gridded surface meteorological data for ecological applications and modelling. *International Journal of Climatology* **33**, 121–131 (2013).

3. Weedon, G. P. *et al.* The wfdei meteorological forcing data set: Watch forcing data methodology applied to era-interim reanalysis data. *Water Resources Research* **50**, 7505–7514 (2014).
